# Assessment of Hydroxyl Radical Reactivity in Sulfur-Containing Amino Acid Models Under Acidic pH

**DOI:** 10.3390/ijms26157203

**Published:** 2025-07-25

**Authors:** Chryssostomos Chatgilialoglu, Piotr Filipiak, Tomasz Szreder, Ireneusz Janik, Gordon L. Hug, Magdalena Grzelak, Franciszek Kazmierczak, Jerzy Smorawinski, Krzysztof Bobrowski, Bronislaw Marciniak

**Affiliations:** 1 Center for Advanced Technologies, Adam Mickiewicz University, Uniwersytetu Poznanskiego 10, 61-614 Poznan, Poland; chachr@amu.edu.pl (C.C.); magdalena.grzelak@amu.edu.pl (M.G.); 2 Istituto per la Sintesi Organica e la Fotoreattivita, Consiglio Nazionale delle Ricerche, Via P. Gobetti 101, 40129 Bologna, Italy; 3 Faculty of Chemistry, Adam Mickiewicz University, Uniwersytetu Poznanskiego 8, 61-614 Poznan, Poland; piotrf@amu.edu.pl (P.F.); kazmak@amu.edu.pl (F.K.); 4 Institute of Applied Radiation Chemistry, Lodz University of Technology, Wróblewskiego 15, 93-500 Lodz, Poland; tomasz.szreder@p.lodz.pl; 5 Institute of Nuclear Chemistry and Technology, Dorodna 16, 03-195 Warsaw, Poland; 6 Radiation Laboratory, University of Notre Dame, Notre Dame, IN 46556, USA; ijanik@nd.edu; 7 Faculty of Medicine and Health Sciences, Kalisz University, Nowy Świat 4, 62-800 Kalisz, Poland; j.smorawinski@uniwersytetkaliski.edu.pl

**Keywords:** methionine, S-methyl-cysteine, pulse and gamma-radiolysis, oxidation, optical absorption, conductivity, free radicals, high-resolution MS/MS

## Abstract

Methionine residues in proteins and peptides are frequently oxidized by losing one electron. The presence of nearby amide groups is crucial for this process, enabling methionine to participate in long-range electron transfer. Hydroxyl radical (HO^•^) plays an important role being generated in aerobic organisms by cellular metabolisms as well as by exogenous sources such as ionizing radiations. The reaction of HO^•^ with methionine mainly affords the one-electron oxidation of the thioether moiety through two consecutive steps (HO^•^ addition to the sulfur followed by HO^−^ elimination). We recently investigated the reaction of HO^•^ with model peptides mimicking methionine and its cysteine-methylated counterpart, i.e., CH_3_C(O)NHCHXC(O)NHCH_3_, where X = CH_2_CH_2_SCH_3_ or CH_2_SCH_3_ at pH 7. The reaction mechanism varied depending on the distance between the sulfur atom and the peptide backbone, but, for a better understanding of various suggested equilibria, the analysis of the flux of protons is required. We extended the previous study to the present work at pH 4 using pulse radiolysis techniques with conductivity and optical detection of transient species, as well as analysis of final products by LC-MS and high-resolution MS/MS following γ-radiolysis. Comparing all the data provided a better understanding of how the presence of nearby amide groups influences the one-electron oxidation mechanism.

## 1. Introduction

The reactive oxygen species (ROS) network acts in various physiological processes [1,2]. Hydroxyl radical (HO^•^) is part of this network, and its harmfulness is connected to the overproduction of ROS [3], and linked with the etiology of various diseases [1,4]. The diffusion distance of HO^•^ is minimal because of its high reactivity with all types of biomolecules, including proteins [5]. Indeed, HO^•^ has long been regarded as a major source of cellular damage [6,7]. The reaction of HO^•^ with methionine residues (Met) in peptides and proteins is a complex multistep process. Although the reaction mechanism has been intensively studied, some essential parts remain unsolved.

The one-electron and two-electron oxidations of sulfide moieties have been intensively studied in peptides and proteins. The reaction of two-electron oxidants like H_2_O_2_, ONOO^−^, or HOCl, produces methionine sulfoxide, Met(O), as the main product [8]. Met(O) exists in two epimeric forms (*S* and *R* epimers) that, in living cells, are explicitly repaired by the enzymes methionine sulfoxide reductase Msr-A and Msr-B, respectively [9,10]. Met in proteins are not only preserved against oxidative stress, but also these transformations play an important role in cellular signaling processes [10,11]. On the other hand, the reaction of HO^•^ with Met formally involves one-electron oxidation through two consecutive steps, i.e., the initial formation of a sulfuranyl radical, followed by heterolytic cleavage, which affords the sulfide radical cation [12,13].

Mechanistic studies on electron transfer (ET) through metal-free peptides and proteins have demonstrated that long-distance ET is possible because several amino acids, including Met, can act as relay stations [14,15]. At first sight it could be surprising that Met can act as a relay amino acid, due to the high reduction potential of dialkyl thioethers (about +1.4 V vs. NHE); however, Met behavior as a relay site can be explained by the neighboring group effect of the adjacent amide function [16]. Indeed, neighboring amide participation in thioether oxidation has been studied in some detail, demonstrating that the stabilization by a neighboring amide group makes Met a target for oxidative stress [17]. Later, pulse radiolysis studies on one-electron oxidation provided evidence for a bond formation between the amide moiety and sulfur in cases where these moieties are juxtaposed [12,13]. It is also worth mentioning that cysteine acts as a relay amino acid, and it has been suggested that the proton transfer during the ET process may be mediated by the surrounding water [15,16].

The ionizing radiation of water is an appropriate way for producing HO^•^ radicals. The technique of pulse radiolysis has provided a myriad of kinetic information concerning reactions involving free radicals (including HO^•^) and biomolecules [18] and, in particular, the radical reactivity of Met in differently functionalized environments [19]. The mechanistic aspects of Met oxidation in various structural environments, as determined by peptide sequences and pH conditions in the presence of one-electron oxidants (including HO^•^), have recently been summarized and discussed [20]. Neighboring group participation appears to be an essential interaction of the sulfide radical cation at the Met residues, controlling the formation of products. Indeed, product characterization and quantification of γ-radiolysis studies on Met [21] and some Met-containing molecules have been reported [22,23,24]. It was established that the formation of small amounts of the matching sulfoxide in all experiments is due to the in situ formation of H_2_O_2_ by the γ-radiolysis of water, rather than to direct oxidation by HO^•^.

**Figure 1:**
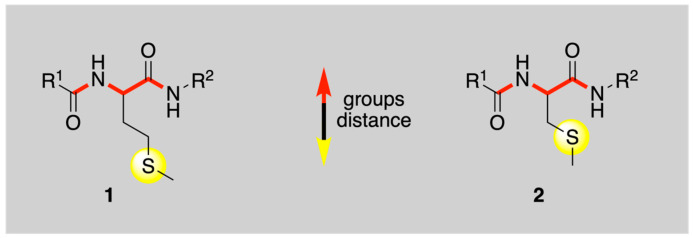
Model peptide backbone (in red) containing methionine residue (**1**) and S-methyl-cysteine residue (**2**) compared in the present oxidation study (R^1^ = R^2^ = Me).

We recently examined the optical detection of transient species by pulse radiolysis and the final products by LC-MS and high-resolution MS/MS after γ-radiolysis for the reaction of HO^•^ with the Met derivative **1** and the cysteine-methylated derivative **2** under anoxic conditions at pH 7 (*vide* Figure 1) [24]. These two compounds contain the simplest model peptide backbone. The comparison between **1** and **2** aimed to understand the influence of the thioether group’s distance from the peptide backbone (**2** has one CH_2_ less) on the chemistry of the HO^•^ radical. A mechanistic scheme was drawn for each derivative and their diversity associated with the type of neighboring amide participation in thioether oxidation. To better understand such differences, it is still necessary to analyze the flux of protons in the various involved equilibria. Herein, we extended the study to pH 4 using the pulse radiolysis approach, with optical detection of intermediate species, and complemented it with the detection of transient conductivity to gain insight into the protic equilibria of the involved intermediates. Additionally, we provided identification and quantification of stable products using LC-MS and high-resolution MS/MS following γ-radiolysis. Comparison of the present results at pH 4 with those at pH 7 afforded a clear mechanistic picture of these reactions.

## 2. Results and Discussion

### 2.1. γ-Radiolysis and Product Analysis at pH 4

Ionizing radiation of neutral water leads to the primary reactive species e^−^_aq_, HO^•^, and H^•^ together with H^+^ and H_2_O_2_ as shown in Reaction 1. The values in brackets represent the radiation-chemical yield (*G*) in µmol J^−1^ [25]. In N_2_O-saturated solution (~0.02 M of N_2_O), e^−^_aq_ are efficiently transformed into HO^•^ radicals via Reaction 2 (*k* = 9.1 × 10^9^ M^−1^ s^−1^) [26], affording *G*(HO^•^) = 0.56 µmol J^−1^. Therefore, HO^•^ and H^•^ account for 90% and 10% of the reactive species, respectively.
H_2_O 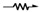 e^−^ _aq_ (0.28), HO^•^ (0.28), H^•^ (0.062), H^+^ (0.28) and H_2_O_2_ (0.073)(1)

e^−^_aq_ + N_2_O + H_2_O → HO^•^ + N_2_ + HO^−^ (2)

We recently reported on LC-MS and high-resolution MS/MS analysis of products generated in neutral (pH 7), N_2_O-saturated, 1 mM aqueous solutions of 1 or 2 upon exposure to 800 Gy of stationary gamma irradiation at a dose rate of 46.7 Gy/min. Herein, we report the analogous experiments performed at pH 4, followed by the same analytical approach. Representative HPLC runs of irradiated samples are shown in the Supporting Material (Figures S1 and S2 for compound **1** and Figures S3 and S4 for compound **2**).

**Figure 2:**
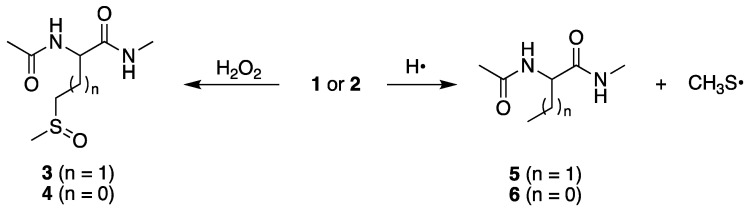
Reactions of methionine (**1**) or S-methyl-cysteine (**2**) derivatives with H_2_O_2_ and H^•^ atom afford the corresponding sulfoxide **3** or **4** and α-aminobutyric (**5**) or alanine (**6**) derivatives, respectively. Methionine series for n = 1 and S-methyl-cysteine series for n = 0.

At pH 7, we reported for compound **1** the formation of sulfoxide **3** and α-aminobutyric derivative **5** in the ratio **3**/**5** = 4.09, and for S-methyl-cysteine derivative **2** the formation of sulfoxide **4** and alanine derivative **6** in the ratio **4**/**6** = 19.28 (Figure 2) [23,24]. These products are also formed at pH 4 in similar ratios, **3**/**5** = 3.82 and **4**/**6** = 26.59, as expected. It is worth underlining that these reactions are not detectable in the pulse-radiolysis study (*vide infra*), therefore their existence is based on the final product identifications. The desulfurization process, with the formation of the methyl thiyl radical (CH_3_S^•^) depicted in Figure 2, is fundamental because some of the products are derived from the combination of CH_3_S^•^ with carbon-centered radicals [27,28].

**Figure 3:**
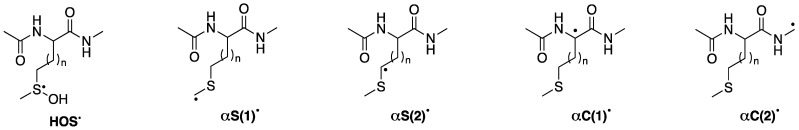
The primary reactions of HO**^•^** with compounds **1** or **2** are the formation of **HOS^•^** adduct and hydrogen atom transfer (HAT) affording four carbon-centered radicals: **αS(1)^•^**, **αS(2)^•^**, **αC(1)^•^**, and **αC(2)^•^**. Methionine series for n = 1 and S-methyl-cysteine series for n = 0.

The reaction of HO^•^ with compound **1** or **2** follows several paths, the main one being the formation of sulfuranyl adduct **HOS^•^** (*vide* Figure 3), which is at the crossroads of various reaction pathways affording a variety of carbon-centered radicals. Previously [24], we found that CH_3_S^•^ couples with the four different carbon-centered radicals to give the corresponding sulfides, and we suggested that the CH_3_S^•^ adducts serve like a footprint of the relative concentration of the four distinct carbon-centered radicals (Figure 3).

Table 1 lists the precursor radicals and product formations for both starting materials at pH 4. Indeed, CH_3_S^•^ reacts with **αS(1)^•^**, **αS(2)^•^**, **αC(1)^•^**, and **αC(2)^•^** for compound **1** and with **αS(1)^•^**, **αS(2)^•^**, and **αC(2)^•^** for compound **2** (Figure 3). For the methionine derivative **1**, the relative concentrations of **αS(2)^•^**, **αS(1)^•^**, **αC(1)^•^**, and **αC(2)^•^** are 12.2/8.6/0.6/1.0, whereas at pH 7 they were reported as 15.4/9.4/1.4/1.0 [24]. The **αS^•^** radicals were the most abundant, being at least one order of magnitude higher than **αC^•^** at both pHs. The amount of **αC(2)^•^** radical increases from pH 7 to pH 4, whereas all the others decrease. In particular, the relative concentrations of two **αC^•^** radicals change considerably, **αC(1)^•^** predominating at pH 7 and **αC(2)^•^** predominating at pH 4. The main source of **αS^•^** being the α-deprotonation of radical cation at sulfur, it is expected for α**S(2)^•^** radical to be found at a higher concentration than α**S(1)^•^** and in line with the higher stability of secondary vs. primary alkyl radical due to favorable deprotonation from the precursor sulfide radical cation. The relative percentage of the two **αS^•^** radicals changes slightly going from pH 7 to 4, as the ratio **αS(2)^•^**/**αS(1)^•^** decreases from 1.64 to 1.42, respectively.

For S-methyl-cysteine derivative **2**, CH_3_S^•^ couples with the carbon-centered radicals **αS(1)^•^**, **αS(2)^•^**, and **αC(2)^•^** to produce the corresponding sulfides. The relative concentrations of **αS(2)^•^**, **αS(1)^•^**, and **αC(2)^•^** are 54.3/1.0/16.6 (Table 1), whereas at pH 7 they were reported as 72.2/1.0/2.3 [24]. The amount of **αC(2)^•^** radical increases going from pH 7 to 4, whereas **αS(2)^•^** decreases. The relative percentages of the two **αS^•^** radicals change slightly going from pH 7 to 4, as the ratio **αS(2)^•^**/**αS(1)^•^** decreases from 72.2 to 54.3, respectively. Moreover, the ratio **αS^•^**/**αC(2)^•^** decreases substantially going from 31.8 at pH 7 to 3.3 at pH 4, suggesting that the acidic conditions favor the formation of **αC(2)^•^** radical.

At pH 4, the high-resolution MS/MS spectra data showed fourteen dimeric compounds obtained from the radiolytic study of starting material **1** (Table S1). The accurate masses of these products (*m*/*z* 407.1781) correspond to a molecular weight MH^+^ equivalent to the coupling of the two carbon-centered radicals (**αS^•^** and/or **αC^•^**). In comparison, sixteen dimeric compounds with *m*/*z* 407.1781 have been obtained at pH 7 [24]. At pH 4, the high-resolution MS/MS spectra data showed five dimeric compounds derived from the radiolytic study of starting material **2** (Table S2). The accurate masses of these products (*m*/*z* 379.1468) correspond to a molecular weight MH^+^ equivalent to the coupling of the two carbon-centered radicals (**αS^•^** and/or **αC(2)^•^**). In comparison, at pH 7, eight dimeric compounds having masses of *m*/*z* 379.1468 are observed [24]. The large majority of these dimeric peaks from compounds **1** and **2** at pH 4 and 7 can be assigned to the same products. These observations align well with the relative concentrations of carbon-centered radicals, which vary at different pHs as discussed in the previous paragraph.

**Figure 4:**
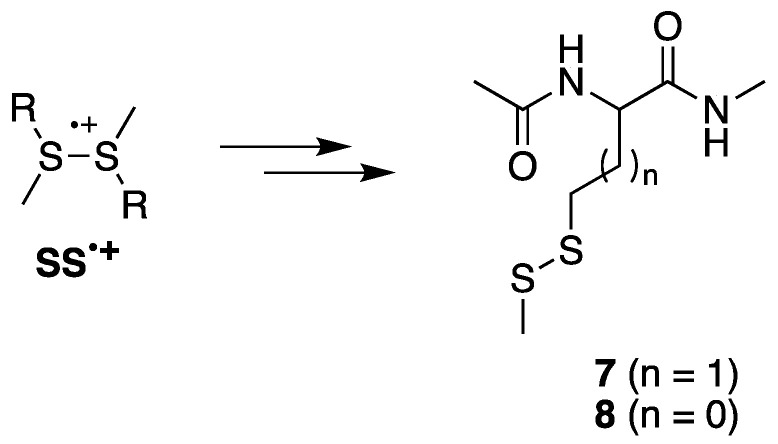
Structure of identified disulfides **7** and **8** observed at both pH 4 and pH 7 based on high-resolution MS/MS spectra. Methionine series for n = 1 and S-methyl-cysteine series for n = 0.

The asymmetric disulfides **7** or **8** are also relevant products derived from the reaction of HO^•^ with compound **1** or **2**, respectively. The sulfide radical cation (**S^•^^+^**) and the starting material (**S**) form an equilibrium with the disulfide radical cation (**SS^•^^+^**). Fragmentation of **SS^•^^+^** affords the observed asymmetric disulfides (Figure 4). The ratios **5**/**7** = 1.28 and **6**/**8** = 0.41 at pH 4, in comparison with the ratios **5**/**7** = 0.67 and **6**/**8** = 0.19 at pH 7, indicate a substantial decrease of **7** and **8** in a more acidic environment.

### 2.2. Pulse Radiolysis in Acidic Environment with Time-Resolved UV–Vis Spectrophotometry and Conductivity

**Figure 5:**
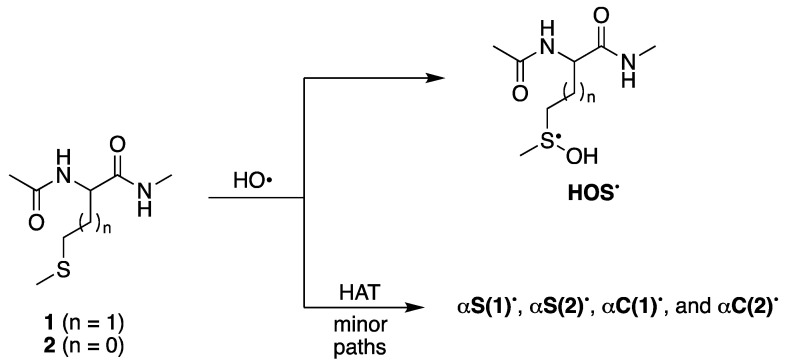
A reaction scheme presents two primary reactions of HO^•^ with **1** or **2** in an aqueous solution, i.e., the formation of HOS^•^ adduct and hydrogen atom transfer (HAT). Methionine series for n = 1 and S-methyl-cysteine series for n = 0.

Pulse radiolysis is the most appropriate technique to study the mechanism of reaction of HO^•^ with sulfur-containing organic compounds [19]. The mechanism of the HO^•^-induced oxidation of sulfur-containing amino acids is based on the addition of the electrophilic HO^•^ radical to the sulfur atom (Figure 5). This was summarized for methionine and its derivatives in a recent review [20], which shows that this reaction is controlled by the diffusion of the reactants and is independent of pH. The second primary reaction, i.e., hydrogen atom transfer (HAT) occurring by HO^•^ radical, is the minor path (<10%) (Figure 5) [29].

The reaction of HO^•^ with compounds **1** and **2** (0.2 mM concentration) in N_2_O-saturated aqueous solutions at pH 4 was studied using pulse radiolysis with the optical absorption detection in a similar way as for neutral solutions (pH 7) [24]. In the current paper, complementary to the optical absorption studies, experiments were conducted using time-resolved conductivity as the detection method [30]. It has been reported in a number of pulse radiolysis studies of simple methionine derivatives and their peptides, that time-resolved conductivity detection can help untangle mechanistic nuances encountered in the analysis of spectral and kinetic changes in optical absorption when multiple transients are formed in the similar spectral range. Their formation can either proceed via the formation of the monomeric sulfur radical cation or by separated coupled electron–proton transfer reactions [31,32,33,34].

**Figure 6:**
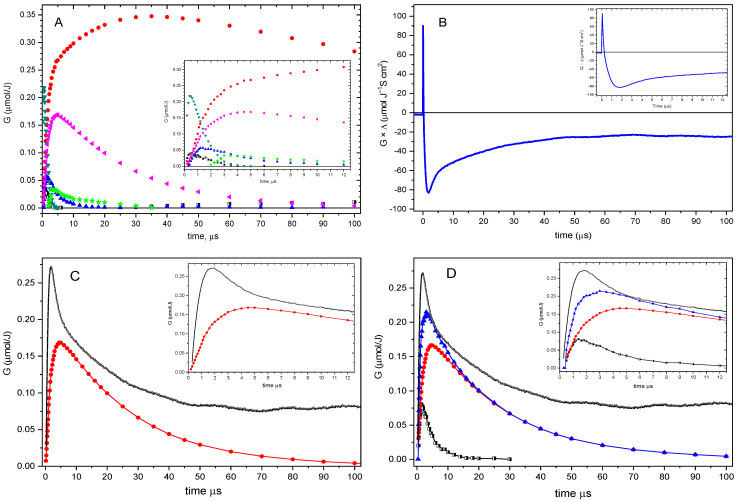
(**A**) Concentration profiles of intermediates formed in the reaction of HO^•^ with **1** obtained using spectral resolution and with the assumption of SN^•^ contribution: αS^•^ (•), SS^•+^ (⏴), SN^•^ (▲), HOS^•^ (▼), αC(1)^•^(■), and αC(2)^•^ (★) in the time range up to 100 μs; inset: in the time range up to 12 μs. (**B**) Equivalent conductivity changes represented as G×ΔΛ vs. time profile following the HO^•^-induced oxidation of **1** in N_2_O-saturated aqueous solution at pH 4 in the time range up to 100 μs; inset: in the time range up to 12 μs. (**C**) Concentration profiles represented as *G*-values of SS^•+^ (•) radicals formed in the reaction of HO^•^ with **1** obtained using spectral resolution (*vide* Figure 6A), and concentration profiles of *G*_ions_ obtained by division of *G*ΔΛ (*vide* Figure 6B) by the overall loss of equivalent conductivity (*vide infra*)––in the time range up to 100 μs; inset: in the time range up to 12 μs. (**D**) Concentration profiles represented as *G*-values of SS^•+^ (•) and SO^•+^ (■) formed in the reaction of HO^•^ with **1** obtained using spectral resolution (*vide* Figure S7), their sum (SS^•+^ + SO^•+^) (▲) and concentration profiles of *G*_ions_ obtained by division of *G*×ΔΛ (*vide* Figure 6B) by the overall loss of equivalent conductivity (*vide infra*)––in the time range up to 100 μs; inset: in the time range up to 12 μs.

Transient absorption spectra in the range 250–700 nm recorded during pulse radiolysis of **1** at pH 4 for time delays in the range 0.6–100 µs are presented in Figure S5A,B. Taking into account known transient spectra of predicted intermediates [24] (Figure S6), one can directly observe from Figure S5A,B the formation and decay of the intermolecular **SS^•^^+^** (at 490 nm) and **αS^•^** (at 290 nm) intermediates for **1**. The obtained transient spectra for various delay times can be analyzed more quantitatively. They can be resolved for a contribution of particular components using the spectral resolution procedure identical to that described previously [22,23,24,29,33]. The results of such a procedure for **1** showing concentration profiles of the most expected intermediates formed in the reaction of HO^•^ with **1** (0.2 mM) are presented in Figure 6A and Figure S7 for pH 4 in N_2_O-saturated aqueous solutions, i.e., **HOS^•^**, **αS^•^**, **SS^•^^+^**, **SN^•^**, **αC(1)^•^**, and **αC(2)^•^** or **HOS^•^**, **αS^•^**, **SS^•^^+^**, **SO^•^^+^**, **αC(1)^•^**, and **αC(2)^•^**, respectively.

**Figure 7:**
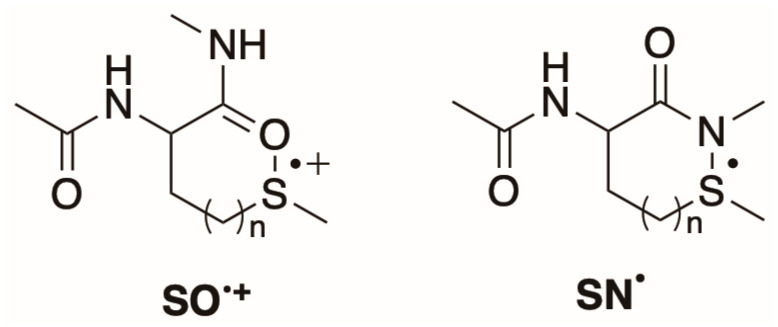
The reactions of compounds **1** or **2** with HO^•^ radical follow several paths affording sulfur–nitrogen three-electron bonded radicals SN^•^ for **1** and **2** and sulfur–oxygen three-electron bonded radical cations SO^+•^ for **2** (*vide* Figure in Section 2.3).

As can be seen from Figure 6A and Figure S7, two main intermediates, **αS^•^** and **SS^•^^+^**, were formed upon the decay of the **HOS^•^** adduct, irrespective of whether **SN^•^** or **SO^•^^+^** (Figure 7) was included in the resolution, yet with a clear dominance of the former.

It is important to note that the comparison of radiation-chemical yields of **SS^•+^** calculated directly from the absorption changes at λ = 490 nm (*vide* Figure S5A,B) with those obtained from the spectral resolutions (*vide* Figure 6A) agrees very well (*vide* Figure S8). There is only a small mismatch in the time region < 10 μs where **SN^•^** and/or **SO^•+^** have only a negligible contribution due to their lower radiation-chemical yields (*vide* Figure 6A and Figure S8), respectively, and much lower molar absorption coefficients at λ = 490 nm (ε_490_) as compared to **SS^•^^+^** (*vide* Figure S6).

Unfortunately, the application of pulse radiolysis with spectral detection, without taking into account results from the conductivity experiments (as it was presented in [24] at pH 7), did not allow to differentiate between **SO^•^^+^** and **SN^•^** transients, since the replacement of **SN^•^** by **SO^•^^+^** in spectral resolutions can still yield similar results (*vide* Figure 6A and Figure S7 for comparison). At pH 7, applying the pulse radiolysis technique with conductivity detection was impossible because the neutralization reaction (*vide infra*) would take too long to reach the base conductivity level after the electron pulse. The application of this technique in an acidic environment could provide additional information on the kinetics and yields of transient ionic species formed during the reaction of HO^•^ with **1** in aqueous solutions. This is based on the fact that the formation of **SS^•^^+^** and **SO^•^^+^** proceeds via the formation of the monomeric sulfur radical cation **S^•^^+^**, and its formation is related to the consumption of protons, reflected in a net decrease in conductivity at acidic conditions. On the other hand, the formation of **SN^•^** proceeds via separated coupled electron–proton transfer reactions involving **HOS^•^** [33]. The HO^−^ generated in the inner-sphere ET, accompanied by **S^•^^+^** formation, would require large solvent reorganization to accommodate two oppositely charged species replacing a neutral **HOS^•^**, as ET precursor. The hydration of the hydroxide anion, whose hydration energy is one of the largest among all anions, would cause the most significant entropic penalty. Therefore, HO^−^ would rather become neutralized in the concerted reaction by the proton released from the neighboring N atom of the amide moiety. This would result in the formation of **S^•^^+^** and **>N^−^**, oppositely charged molecular fragments separated by the -OH moiety of a freshly formed water molecule. Upon the attraction of two oppositely charged fragments, the water molecule becomes squeezed out, and a new hemibonded **SN^•^** transient is formed, showing an apparent transient absorption signal but no evident net change in conductivity [33]. The results of these studies at pH 4 are shown in Figure 6B–D.

After electron pulse in N_2_O-saturated acidic solution (pH = 4) containing 0.2 mM of **1**, an instantaneous growth of the equivalent conductivity was observed followed by its fast decrease below the conductivity level recorded before the pulse, reaching the minimum at 2 μs after the pulse and followed by its slow increase for the next 100 microseconds (Figure 6B). The initial transient conductivity spike is a result of a net increase in conductivity due to the production of conducting species of water radiolysis (hydrated electrons (e^−^_aq_) and protons (H^+^)). In N_2_O-saturated aqueous solutions, e^−^_aq_ is quickly converted into HO^•^ radicals, with the side product of HO^−^ anions being released as well within just a few nanoseconds after the electron pulse. Therefore, the fast decrease in conductivity is recorded within less than 1 μs after the pulse, through a stoichiometric neutralization reaction (H^+^ + HO^−^ → H_2_O) with (1.1–1.2) × 10^11^ M^−1^ s^−1^ [35,36], involving highly conducting HO^−^ and H^+^ ions. This was confirmed by the observation of the relaxation of ionic conductivity in pulse-irradiated N_2_O-saturated pure water at pH 4 (*vide* Figure S9). Upon completion of this reaction, the conductivity level further decreases, since HO^−^ anions formed in reaction leading to the monomeric sulfur radical cations (**S^•^^+^**) removes H^+^, and consequently, highly conducting protons (H^+^) (Λ = 350 S cm^2^) are replaced by weakly conducting sulfur radical cations (**SS^•^^+^**) and/or (**SO^•^^+^**) (Λ = 45 S cm^2^) (*vide* Figure 6B). Thus, the overall loss of equivalent conductivity can be calculated as ΔΛ_0_ = −305 S cm^2^. The conductivity data not only provide kinetic data but also allow for an estimation of the radiation-chemical yields of **S^•^^+^** and, consequently, the radiation-chemical yields of **SS**^•^**^+^** and/or **SO^•^^+^**. The radiation-chemical yields of ions (*G*(ions)) can be calculated from the experimental changes in equivalent conductivity at given times (*G* × ΔΛ) (*vide* Figure 6B). Dividing these values by ΔΛ_0_ = −305 S cm^2^ gave *G*(ions) at given times presented in Figure 6C,D. A comparison of the radiation-chemical yields of **SS^•^^+^** and the sum of **SS^•^^+^ + SO^•^^+^** obtained from the resolution of absorption spectra and *G*(ions) is presented in Figure 6C and D, respectively.

At this point, it is important to note that neither the inclusion of **SN^•^** while leaving out **SO^•^^+^**, nor inclusion of **SO^•^^+^**, while leaving out **SN^•^** in spectral resolutions, gives *G*(**SS^•^^+^**) or the sum of *G*(**SS^•^^+^** + **SO^•^^+^**), respectively, that matches *G*(ions) as determined by conductivity measurements. In the first case, the significant discrepancy between *G*(ions) = 0.27(5) μmol J^−1^ and *G*(**SS^•^^+^**) = 0.12(5) μmol J^−1^ occurs at 2 μs after the pulse (*vide* inset in Figure 6C). In the second case, this discrepancy is less pronounced: *G*(ions) = 0.27(5) μmol J^−1^ vs. the sum of *G*(**SS^•^^+^** + **SO^•^^+^**) = 0.20 μmol J^−1^ (*vide* inset in Figure 6D). Therefore, the only possible intermediate responsible for the observed loss of conductivity (higher than would result from the radiation-chemical yields of **SS^•^^+^** and **SO^•^^+^** obtained from the resolution of absorption spectra) is **S^•^^+^**, which was not included in the spectral resolutions. Furthermore, the rapid increase in equivalent conductivity in the range of 2 to 10 microseconds can be explained by its deprotonation leading to the **αS^•^** (*vide* inset in Figure 6B).

Another important issue is to make a conclusive decision on which of the **SN^•^** and **SO^•^^+^** transients is responsible for the absorption band located in the range of 390–400 nm. The results obtained from the time-resolved conductivity would indicate the involvement of **SO^•^^+^** rather than **SN^•^**; however, they are not clearly convincing. This raises another question: is there a process by which these two transient species can be ultimately distinguished? Based on the known sulfur radical chemistry derived from research on the oxidation of peptides containing methionine and S-methylcysteine [33,37], the decay mechanisms of **SO^•^^+^** and **SN^•^** should allow them to be distinguished. The mechanism of **SN^•^** decay, related to the ring opening, involves protons leading, inter alia, to **S^•^^+^**. In turn, the mechanism of **SO^•^^+^** decay, related to the ring opening, involves only a dynamic equilibrium between **SO^•^^+^** and **S^•^^+^**, without participation of protons, followed by **S^•^^+^** deprotonation. In other words, the rate of **SN^•^** decay, unlike the rate of **SO^•^^+^** decay, should be pH-dependent.

**Figure 8:**
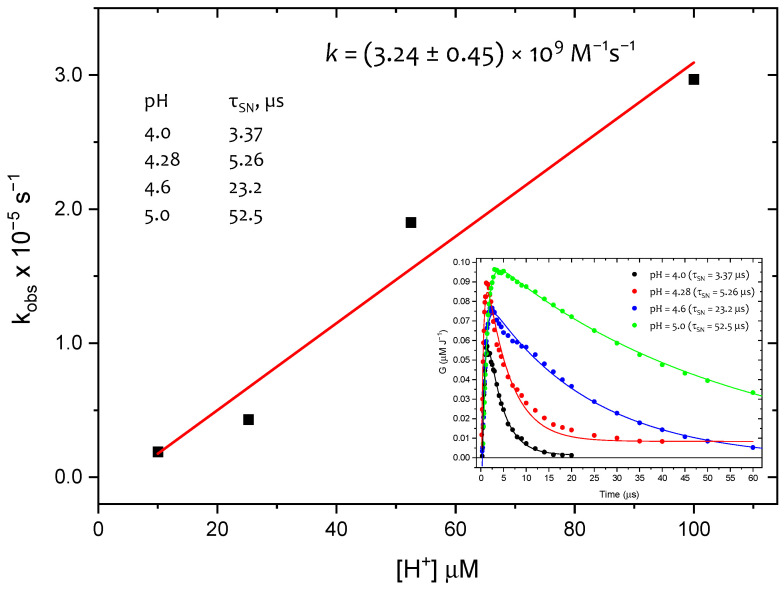
Dependence of k_obs_ for the decay of SN^•^ on [H^+^] concentration. Inset: concentration profiles for SN^•^ represented as radiation chemical yields, (G) vs. time, at various pH values: 4.0 (•), 4.3 (•), 4.6 (•), and 5.0 (•). Decays of SN^•^ were fitted using the formula *G* = *G*_0_ exp(-k_obs_t) + *G*_lim_.

The resolutions of absorption spectra at additional pH values of, 4.3, 4.6, and 5.0, yielded concentration profiles of SN^•^ (inset in Figure 8).

As expected, the decay rate of **SN^•^** was pH-dependent, increasing with higher proton concentrations (inset in Figure 8). From the plot of the pseudo-first-order rate constants versus proton concentration (Figure 8), the rate constant *k*(SN^•^ + H^+^) was determined to be (3.2 ± 0.5) × 10^9^ M^−1^ s^−1^. This *k*-value is in very good agreement with the *k*-value determined for the same reaction in cyclic dipeptide *c*-(L-Met-L-Met) and equal to *k*(SN^•^ + H^+^) = (2.1 ± 0.1) × 10^9^ M^−1^ s^−1^ [33]. For comparison, using the same spectral resolution approach but with **SO^•^^+^**, instead of **SN^•^**, a similar picture was obtained (*vide* Figure S10). This fact clearly indicates the inconsistency of these results with the expected independence of the **SO^•^^+^** decay rate from the proton concentration, and thus confirms the involvement of **SN^•^** in the oxidation mechanism of **1** by HO^•^. With this information in hand, the phenomenon of the increasing mismatch between *G*(**SS^•+^**) and the *G*(ions) with increasing time after the electron pulse (Figure 6C) requires explanation.

As it was mentioned earlier, the decay of the conductivity signal, that is, an increase in acid solutions after reaching the negative extreme value, is indicative of a liberation of protons from **S^•^^+^** (*vide* inset in Figure 6B). This first step in the recovery of the conductivity signal occurring during the first 7 μs after the electron pulse coincides very well with the first step of formation of **αS^•^** radicals and is attributed to the deprotonation of **S^•^^+^** (*vide* inset in Figure 6A). Then, the persistent constant difference between *G*(ions) and *G*(**SS^•^^+^**), observed in the time domain up to 12 μs (*vide* inset in Figure 6C), is consistent with the **SN^•^** decay (*vide* inset in Figure 8) and is attributed to the presence of **S^•^^+^** and **NH^•^^+^** radical cations, which are formed upon protonation of **SN^•^**. Interestingly, the recovery of the conductivity signal is similar to the decay of **SS^•^^+^**. This observation can be rationalized as follows: though protons are consumed during the protonation of **SN^•^**, the transients formed in this reaction, i.e., S**^•^**^+^ and NH**^•^**^+^ radical cations, undergo rapid deprotonation. The occurrence of these two reactions causes no net change in conductivity. However, with the further elapse of time, a difference between the kinetics of **SS^•^^+^** decay and the kinetics of recovery of the conductivity signal becomes more and more pronounced. The conductivity signal uptake up to 100 μs, plateauing between 50 and 100 μs (even though **SS^•^^+^** optical signal decays to nearly zero in the same time) (*vide* Figure 6C), has to be explained by the secondary processes in which protons are consumed. This suggestion is based on similar findings in the oxidation of simple thioethers [38], as well as cyclic 1,3-dithiane [39], and 1,3,5-trithiane compounds [40]. As for the moment, the most reasonable explanation seems to be disproportionation of **αS^•^**, resulting in the restored original compound **1** and formation of sulfur cation (**S^+^**) (*vide* Figure S11).

The **αS^•^(2)**, which can be described in two mesomeric forms, undergoes a disproportionation reaction that leads to negative [–CH=S^−^–CH_3_] and positive ions [–CH=S^+^–CH_3_]. The negative ions, which can also exist in another mesomeric form [–C^−^H–S–CH_3_], will presumably pick up a proton, yielding the original compound **1**. This reaction reflects the substitution of a proton (H^+^) by a positive ion [–CH=S^+^–CH_3_], resulting in a negative conductivity signal that persists up to 100 μs. The plateau section is compatible with the expected long lifetime of a positive ion (S^+^).

There is one more observation that needs to be explained, namely the high radiation chemical yield of **αS^•^** (*G* = 0.27 μmol J^−1^) at 4 μs after the electron pulse. Direct abstraction of the hydrogen atom by HO^•^ cannot be responsible because the contribution of this reaction is only 10% and can lead to the formation of **αS^•^** with a maximum radiation chemical yield, *G* = 0.06 μmol J^−1^. Similarly, the deprotonation of **S^•^^+^** within this time range does not fully account for the high G-value of **αS**^•^. Based on the concentration profiles of *G*_ions_ (*vide* inset in Figure 6C), the radiation chemical yield of **αS^•^** formed via this reaction is nearly equal to *G* = 0.05 μmol J^−1^. Simple calculation indicates that the remaining 0.16 μmol J^−1^ of **αS^•^** must be formed in another competing process, which is not associated with the change in conductivity. By analogy with the formation of **SN^•^**, the formation of **αS^•^** must also proceed via separated coupled electron–proton transfer reactions involving **HOS^•^**. The HO^−^ generated in the inner-sphere electron transfer that leads to **S^•^^+^** is neutralized in the concerted reaction by the proton released at C atoms located in methylene and/or methyl groups adjacent to the S atom, leading to the formation of either **αS(2)^•^** or **αS(1)^•^** radicals, respectively. An analogous reaction was proposed earlier for simple aliphatic sulfides [38], and tetrahydrothiophene [36].

Table 2 summarizes the radiation chemical yields of all transients formed from **1** at selected times when one of the transients reaches its maximum yield.

Next, we will discuss the results for compound **2**. Transient absorption spectra in the range 250–700 nm recorded during pulse radiolysis of **2** at pH 4 for time delays in the range 0.6–100 µs are presented in Figure S12A,B.

**Figure 9:**
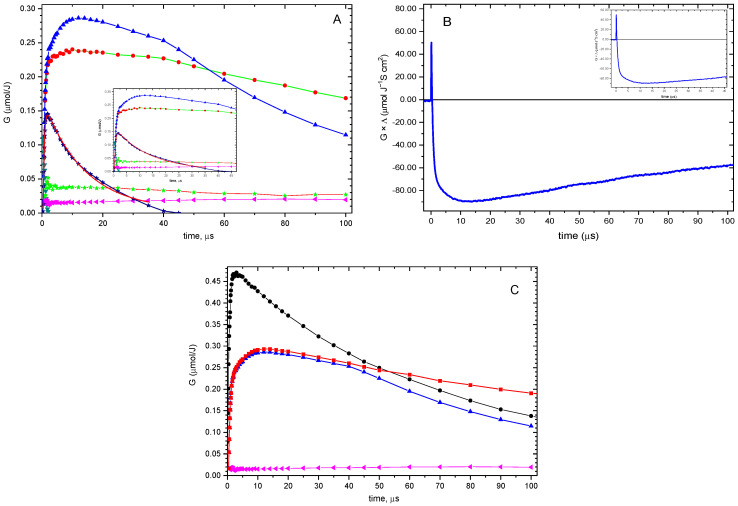
(**A**) Concentration profiles of intermediates formed in the reaction of HO^•^ with **2** obtained using modified spectral resolution combined with conductivity (*vide* text) and with the assumption of SN^•^ contribution: αS^•^ (•), SS^•+^ (⏴), SO^•+^ (▲), SN^•^ (★) HOS^•^ (▼), and αC(2)^•^ (★) in the time range up to 100 μs; inset: in the time range up to 47 μs. (**B**) Equivalent conductivity changes represented as G×ΔΛ vs. time profile following the HO^•^-induced oxidation of **2** in N_2_O-saturated aqueous solution at pH 4 in the time range up to 100 μs; inset: in the time range up to 47 μs. (**C**) Concentration profile represented as *G*-values of SO^•+^ formed in the reaction of HO^•^ with **2** obtained from the absorbance at λ = 390 nm, assuming that SO^•+^ was the only transient responsible for the absorption (•); concentration profile represented as *G*-values of ions formed in the reaction of HO^•^ with **2** obtained by division of G×ΔΛ (Figure 9B) by the overall loss of equivalent conductivity (■); concentration profiles represented as *G*-values of SO^•+^ (▲) and SS^•+^ (⏴) formed in the reaction of HO^•^ with **2** obtained using modified spectral resolution (*vide* Figure S13).

From the comparison of these spectra with those of compound **1** (*vide* Figure S5A,B), it is evident that the **SS^•^^+^** intermediate for **2** is nearly absent, the yield of **αS^•^** is lower, and a strong, distinct absorption band with λ_max_ = 390 nm appears. Similarly to **1**, the high radiation chemical yield of **αS^•^** (*G* = 0.22 μmol J^−1^) at 2.5 μs after the electron pulse can be rationalized by a separated coupled electron–proton transfer reaction involving **HOS^•^**. The question arises as to which transient species are responsible for the absorption with a maximum located at λ = 390 nm, given the expected presence of **SO^•^^+^** and/or **SN^•^** transients. In our previous paper, we assigned this absorption band only to **SO^•^^+^** [24]. Fortunately, the pulse radiolysis technique, combined with conductivity detection, played a crucial role in reaching the final conclusion on this issue. Assuming that **SO^•^^+^** was the only transient responsible for the absorption, the radiation-chemical yield of **SO^•^^+^**, calculated directly from the absorption change at λ = 390 nm and assuming ε_390_ = 3009 (*vide* Figure S6), measured at 1.8 μs, was found to be equal to 0.47 μM J^−1^ (*vide* Figure 9C). On the other hand, the *G*(ions) at 1.8 μs calculated from the experimental changes in equivalent conductivity at given times (*G* × ΔΛ) (*vide* Figure 9B) were found to be equal to 0.23 μM J^−1^ (*vide* Figure 9C).

Since **SO^•^^+^** is the only possible intermediate responsible for the observed loss of conductivity at 1.8 μs, it is obvious that **SO^•^^+^** cannot be the only transient responsible for absorption with λ_max_ = 390 nm. The obvious candidate is **SN^•^**, which is characterized by an identical shape and location of the absorption band; however, it has a higher molar absorption coefficient compared to **SO^•+^** (*vide* Figure S6). Therefore, the usual procedure for resolving experimental transient spectra cannot be applied to compute their initial radiation-chemical yields reliably. To achieve this, a slightly modified spectral resolution procedure, combined with conductivity measurements, was employed. The radiation-chemical yield of **SO^•^^+^** at 1.8 μs can be measured independently from the conductivity (*G* = 0.23 μM J^−1^, vide supra), so their contribution in the resulting experimental spectrum *G*(SO^•+^) × ε(SO^•+^)_390_ can be easily determined and subtracted from the experimental spectra. Subsequently, the spectrum resulting from that subtraction was decomposed into the component spectra associated with the various transient species present (**HOS^•^**, **SN^•^**, **αS^•^**, **SS^•^^+^**, and **αC(2)^•^**) except **SO^•^^+^**, using the spectral resolution procedure identical to that described previously [24] (see also Material and Methods section). At this step of the procedure, the most important parameter is the radiation-chemical yield of **SN^•^** at the maximum of its formation (G_SN_^•^ = 0.14 μM J^−1^, vide supra), which is taken later as an initial concentration in its decay. Our previous experiments with the cyclic peptide *c*-(Met-Met) have demonstrated partial conversion of **SN^•^**, involving proton participation, into the intramolecular dimeric radical cation **SS^•^^+^**. Based on this observation, it was assumed that a similar type of reaction might occur in compound **2**, in which **SN^•^** undergoes proton-dependent conversion into **SO^•^^+^**. The *G*(SN**^•^**) determined from these decays for selected time points was used to calculate their contribution to the resulting experimental spectrum *G*(SN^•^) × ε(SN^•^)_390_, and was further subtracted from the experimental spectra. Subsequently, the spectrum resulting from that subtraction was decomposed into the component spectra associated with the various transient species present (**HOS^•^**, **SO^•^^+^**, **αS^•^**, **SS^•^^+^**, and **αC(2)^•^**) except **SN^•^.** The rate constant of this reaction was taken as a parameter, and using the trial-and-error method, the optimum rate constant for **SN^•^** decay was adjusted, until a concentration profile of **SO^•^^+^** for an initial period of its decay was similar to a concentration profile of ions determined by conductivity (*vide* Figure S13). Thus, the determined pseudo first-order rate constant for the protonation of **SN^•^** was found to be equal to 7 × 10^4^ s^−1^ (*vide* Figure 9C), which roughly corresponds to a second-order rate constant *k* = 7 × 10^8^ M^−1^ s^−1^. This value is lower than the rate constant for the **SN^•^** protonation reaction of **1** (vide supra). It is worth emphasizing that pulse radiolysis experiments, which included conductivity and spectral detections, enabled the determination of concentration profiles of **SO^•^^+^** formed simultaneously with **SN^•^** during ^•^OH-induced oxidation of **2.** Similarly to 1, with the further elapsed time after completion of **SN^•^** decay, a difference between the kinetics of **SO^•^^+^** decay and the kinetics of recovery of the conductivity signal becomes increasingly pronounced. Just as for **1**, this observation can be explained again by a set of reactions starting with the disproportionation of **αS^•^**, resulting in the restoration of the original compound **2** and formation of the respective sulfur cation (**S^+^**) (*vide* Figure S11).

Table 3 summarizes the radiation-chemical yields of all transients formed from **2** at selected times when one of the transients reaches its maximum yield.

### 2.3. Mechanistic Insights

**Figure 10:**
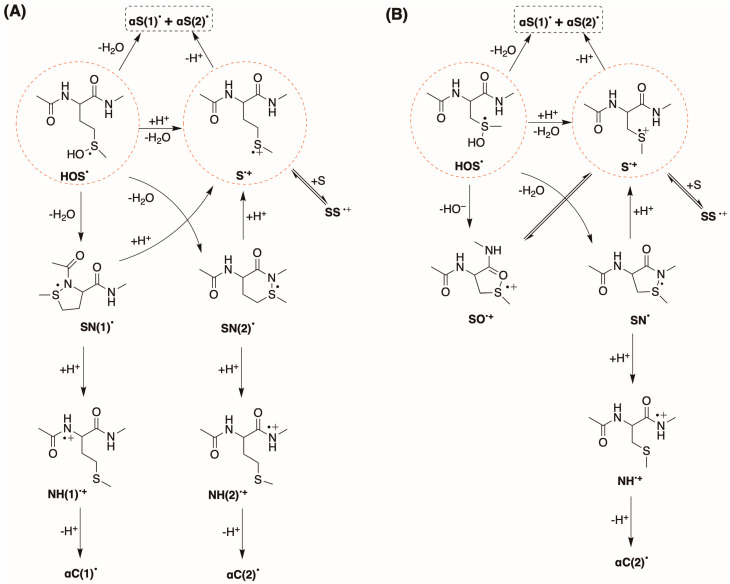
Proposed mechanisms for the reaction of HO^•^ radicals with methionine derivative **1** (**A**) and cysteine derivative **2** (**B**) starting from their corresponding HOS^•^ adducts (*vide* Figure 5).

The radiation-chemical yields (G) of the primary reactive species (e^−^_aq_, HO^•^, and H^•^) are the same in the pH range of 4–7 (*vide* Reaction 1); therefore, the differences only apply to those processes involving protons (H^+^) from the bulk of solution. It is well known that the reaction of HO^•^ with Met, and, in general, with dialkyl sulfides, occurs mainly by addition to the sulfur atom, generating a sulfuranyl radical (**HOS^•^**). With Met, the direct H-atom abstraction from the C—H bonds is a minor path that accounts for less than 10% (*vide* Figure 5). Figure 10A,B summarizes the fate of **HOS^•^** for compounds **1** and **2**, respectively. In both cases, the **HOS^•^** eliminates HO^−^ very fast which is subsequently neutralized either by external protons (H^+^) and affording the one-electron oxidation of the thioether moiety (**S^•^^+^**) or by protons released either at the C atoms located in methylene and/or methyl groups adjacent to the S atom, leading to the formation of either **αS(2)^•^** or **αS(1)^•^**, respectively. Additionally, the **HOS^•^** eliminates HO^−^, which is neutralized by protons released at the N-atoms located on both sides of the Met moiety, leading to the formation of **SN(1)^•^** and **SN(2)^•^** for compound **1**. Part of **S^•^^+^** undergoes deprotonation, affording the formation of α**S^•^(1)** and α**S^•^(2)** radicals, and the latter is a precursor of **SS^•^^+^** in the case of **1** and **SO^•^^+^** in the case of **2**.

The **αS^•^** radicals account for ca. 50% in **1** (*vide* Table 2) and ca. 35% in **2** (*vide* Table 3) of all radicals present after full decay of **HOS^•^**. Comparison of the respective contribution of **αS^•^** obtained at pH 7 [24] clearly shows that deprotonation of S^•+^ is an additional source of them beyond the process of their formation directly from **HOS^•^** (vide supra). From product studies, the ratio **αS(2)**^•^/**αS(1)^•^** is ca. 1.5 for **1** in both pH 4 and 7, whereas for **2** is 72.2 and 54.3 at pH 7 and 4, respectively. Therefore, the deprotonation of an internal –CH_2_S– is much faster than the external –SCH_3_ in **2**, probably due to conformational preference and/or to higher radical stabilization of **αS(2)^•^**.

The formation of **SS^•+^** for **1** is an important reaction path, as further confirmed by the conductivity experiments and formation of disulfide **7** (*vide* Figure 4). The **SS^•+^** radicals account for ca. 35% (at the maximum of their formation) of all radicals present after full decay of **HOS^•^** in **1** (*vide* Table 2). On the other hand, the contribution of **SS^•+^** is minor, as evident from the formation and decay of transients in Figure S12A,B for **2**. Their contribution accounts for ca. 3% of all radicals present (*vide* Table 3). It is worth mentioning that disulfide **8** is a reaction product of **2**, confirming the **SS^•+^** reaction path, but the yield is approximately four-fold lower than that of the corresponding product 7 in both pH 4 and 7 (*vide* Figure 4).

The five- and/or six-membered **SN^•^** species containing 2c-3e bonds are well documented [33]. In **1**, there is a possibility to obtain both five-membered **SN(1)^•^** and six-membered **SN(2)^•^** species by the left and right interactions of the sulfur atom with N-atoms in the peptide backbone. It has to be stressed that the formation of both **SN^•^** species does not occur via the **S^•^^+^** pathway but involves their direct formation from **HOS^•^** via separated coupled electron–proton transfer reactions (vide supra). Their contribution is not very high and accounts for ca. 13% of all radicals present at the time of their maximum yield (*vide* Table 2). It is well documented that **SN^•^** species undergo fast reaction with protons (H^+^) [33]. In **1**, the protonation of both, **SN(1)^•^** and **SN(2)^•^**, can lead either to **S^•^^+^** which further deprotonate to **αS^•^** or to **NH(1)^•^^+^** and **NH(2)^•^^+^**, which further deprotonate, yielding **αC(1)^•^** and **αC(2)^•^** radicals, respectively. The transient absorption spectra of SN^•^ do not distinguish the latter two reaction paths. However, these two distinct pathways are confirmed by the product studies, where the ratio CH_3_S—αC(1)/CH_3_S—αC(2) is 0.6/1 at pH 4 and 1.4/1 at pH 7, thus the acidic environment favoring the formation of **αC(1)^•^** respect to **αC(2)^•^**.

One more issue requires comment, namely, the absence of **SO^•+^** in the reaction scheme for **1**. Analyzing the structure of **1**, the cyclic **SO^•+^** formed will be six- or seven- membered. As reported previously [41], formation of six-membered **SO^•+^**, though thermodynamically possible, is not able to compete kinetically with deprotonation of its immediate precursor, i.e., S^•+^. A similar argument can be applied to the seven-membered **SO^•+^**. Moreover, supporting the above conclusions, their presence was not necessary to explain the experimental observations.

Interestingly, in the case of **2**, **αC(1)^•^** is not formed in the acidic environment, similar to the experiments at pH 7 (*vide* Figure 9A and Table 3). This is in agreement with the results from the analysis of the stable products, where products containing **αC(1)^•^** are not detected (*vide* Table 1). The lack of **αC(1)^•^** can be rationalized by the absence of **SN^•^** (cyclic four-membered **SN** radical with N-atom from N-acetyl group of N-terminal) and consequently NH**^•^^+^** as its precursor. It has to be stressed that the contribution of **SN^•^** and **SO^•^^+^** accounts for ca. 21% and 45% of all radicals present at the time of their maximum yield (*vide* Table 3). This is not surprising, considering that both transients are characterized by a five-membered cyclic structure. It is also worth underlining that the ratio CH_3_S—αS/CH_3_S—αC(2) decreases substantially going from 31.8 at pH 7 to 3.3 at pH 4, suggesting that the acidic conditions favor the formation of **αC(2)^•^**. Figure 10A,B summarize the fate of HOS^•^ species for compounds **1** and **2**.

Both starting compounds **1** and **2** have **αS^•^** radicals as one of the main intermediates, although the ratio **αS(2)^•^**/**αS(1)^•^** changes substantially from **1** to **2** (*vide* Table 1). Other important intermediates are the following: **SS^•^^+^** for **1** and **SN^•^** and **SO^•^^+^** for **2**. In turn, the contribution of **αC(1)^•^** and **αC(2)^•^** for **1** and **αC(2)^•^** for **2** is rather minor (*vide* Table 2 and Table 3, respectively). However, in relation to **αS(2)^•^** radicals, contribution of **αC(2)^•^** radicals for **2** compared to **1** is higher which was confirmed by the ratio CH_3_S—αS(2)/CH_3_S—αC(2) equal 12.2/1 for **1** and 54.3/16.6 for **2**, respectively (*vide* Table 1).

## 3. Materials and Methods

The compounds **1** and **2** were synthesized according to the procedure described in the Supporting Information of Ref. [42].

Steady-state γ-radiolysis and LC-MS/MS measurements were performed as described previously in Ref. [24].

### 3.1. Pulse Radiolysis

Pulse radiolysis experiments with spectral detection were performed with the LAE-10 electron accelerator and pulse radiolysis setup at the Institute of Nuclear Chemistry and Technology in Warsaw, Poland, as described in [43]. Absorbed doses per pulse were on order of 11Gy (1 Gy = 1 J kg^−1^). The dosimetry was based on N_2_O-saturated solutions of 10^−2^ M KSCN, which, following radiolysis, produces (SCN)_2_^•−^ that are characterized by a molar absorption coefficient of 7580 M^−1^ cm^−1^ at λ = 472 nm, and are produced with a yield of *G* = 0.635 μmol J^−1^ [44].

Pulse radiolysis experiments with conductivity detection were performed with the Titan 8 MeV Beta model TBS 8/16-1S linear accelerator at Notre Dame Radiation Laboratory, USA. The conductivity setup for time-resolved conductivity measurements was used. It allows high-precision conductometric measurements over a pH range from 3 to 6. In the current experiments, pH was restricted from 4 to 5. A detailed description of the conductivity setup, the measuring cell concept, workflow, and data processing was given elsewhere [35]. The current studies employed two newer variants of the conductivity cell, featuring different cell constants and dead volumes, along with an upgraded detection setup equipped with a 12-bit oscilloscope (LeCroy HDO6104A) and data acquisition/control software. The dosimetry was achieved using an acidic (pH = 4.1) aqueous solution saturated with methyl chloride (CH_3_Cl). In this dosimeter system, pulse irradiation yields H^+^ and Cl^−^ with G(H^+^) = G(Cl^−^) = 0.285 μmol J^−1^. The respective equivalent conductivities at 18 °C were taken as Λ(H^+^) = 315 S cm^2^ equiv^−1^ and Λ(Cl^−^) = 65 S cm^2^ equiv^−1^ [45].

### 3.2. Spectral Resolutions of Transient Absorption Spectra

The spectral resolutions of transient absorption spectra at various time delays following the electron pulse into individual components were performed by applying linear regression analysis according to the following equation:

*G*ε(λ_ι_) = Σ ε_j_(λ_i_) *G*_j_ (3)

where ε_j_ is the molar absorption coefficient of the j-th species and the regression parameters, *G*_j_, are equal to the radiation-chemical yield of the j-th species. The sum in Equation (3) is over all radical species present. For any particular time-delay of an experiment, the regression analysis included equations such as Equation (3) for each λ_i_ under consideration. Thus, the spectral resolutions were made using Equation (3) by fitting the reference spectra to the observed transient spectra, transformed from OD(λ) to Gε(λ) using the dosimetry described above. Further details of this method were described elsewhere in [24,33]. The reference spectra of the relevant transient were previously collected and applied in the spectral resolutions (*vide* Figure S6 in Supporting Material) [33].

## 4. Conclusions

We performed detailed studies on HO^•^-induced oxidation of methionine and its S-methyl-cysteine analog using a simplified peptide structure which mimics their location in the interior of either a peptide or protein molecule. We performed the experiments in an acidic environment (pH = 4) to track the role of protons (H^+^) during the reaction, but also to enable the examination of this system using the time-resolved conductivity method. This study expands upon previous work conducted at pH 7 [24]. By combining multiple experimental techniques, we were able to map out the detailed reaction pathways for both compounds, as shown in Figure 10. Our findings revealed that these two compounds react differently upon oxidation by HO^•^ radicals. The results suggest that neighboring amide groups, with different atoms involved, participate in the reaction, and the process includes various equilibria involving short-lived intermediates that form five- and/or six-membered ring structures.

## Figures and Tables

**Table 1:** Product formations by combination of CH_3_S^•^ ^a^ with carbon-centered radicals at pH 4 ^b,c^.

Products	Compound 1 ^d^	Compound 2 ^d^
CH_3_S—αS(2)	12.2	54.3
CH_3_S—αS(1)	8.6	1
CH_3_S—αC(1)	0.6	—
CH_3_S—αC(2)	1	16.6

^a^ Generated from the reactions of H^•^ atoms—see Figure 2; ^b^ N_2_O-saturated solution of **1** or **2** (1.0 mM) irradiated for 800 Gy (46.7 Gy/min); ^c^ based on LC-MS and high-resolution MS/MS analysis; ^d^ based on the intensity of LC peaks at pH 4.

**Table 2:** The radiation chemical yields (*G*, μmol J^−1^) of radicals and their percentage contribution (in parentheses) to the total yield of radicals present in the reaction of HO^•^ with **1** at different times after the electron pulse at pH 4.

Time (μs)	HOS^•^	αC(1)^•^	αC(2)^•^	SN^•^	SS^•+^	αS^•^	Total R^•^
0.4	**0.22 ^a^** **(68.8%)**	**0.04 ^a^** **(12.5%)**	0	0.02 (6.3%)	0.03 (9.4%)	0.01 (3.0%)	0.32
1.4	0.12 (25.5%)	0.03 (6.4%)	0	**0.06 ^a^** **(12.8%)**	0.11 (23.4%)	0.15 (31.9%)	0.47
3.0	0.01 (2%)	0.01 (2%)	**0.04 ^a^** **(8%)**	0.05 (10%)	0.15 (30%)	0.24 (48%)	0.50
4.5	0	0	0.03 (6%)	0.03 (6%)	**0.17 ^a^** **(34%)**	0.27 (54%)	0.50
10	0	0	0.02 (4.2%)	0.01 (2.1%)	0.15 31.3%)	0.30 (62.5%)	0.48
35	0	0	0	0	0.06 (14.6%)	**0.35 ^a^** **(85.4%)**	0.41
100	0	0	0	0	0.01 (3.4%)	0.28 (96.6%)	0.29

**^a^** the highest radiation chemical of the transient determined from the spectral resolutions.

**Table 3:** The radiation-chemical yields (*G*, μmol J^−1^) of radicals and their percentage contribution (in parentheses) to the total yield of radicals present in the reaction of HO^•^ with **2** at different times after the electron pulse at pH 4.

Time (μs)	HOS^•^	αC(2)^•^	SN^•^	SO^•+^	SS^•+^	αS^•^	Total R^•^
0.4	**0.11 ^a^** **(44%)**	0	0.04 (16%)	0.05 (20%)	0	0.05 (20%)	0.25
1.8	0.01 (1.5%)	**0.04 ^a^** **(6%)**	**0.14 ^a^** **(21.2%)**	0.23 (34.8%)	**0.02 ^a^** **(3%)**	0.22 (33.3%)	0.66
10	0	0.04 (6.1%)	0.08 (12.3%)	0.28 (43.1%)	0.02 (3.1%)	**0.23 ^a^** **(35.4%)**	0.65
14	0	0.04 (6.3%)	0.06 (9.3%)	**0.29 ^a^** **(45.3%)**	0.02 3.2%)	0.23 (35.9%)	0.64
50	0	0.04 (7.8%)	0	0.23 (45.1%)	0.02 (3.9%)	0.22 (43.1%)	0.51
100	0	0.03 (9.1%)	0	0.11 (33.3%)	0.02 (6.1%)	0.17 (51.5%)	0.33

**^a^** the highest radiation chemical of the transient determined from the spectral resolutions.

## Data Availability

All data are displayed in the manuscript and Supporting Material.

## Supplementary Materials

The following supporting information can be downloaded at: https://www.mdpi.com/article/10.3390/ijms26157203/s1.
