# Supplementary material for: Assessment of Hydroxyl Radical Reactivity in Sulfur-Containing Amino Acid Models Under Acidic pH
Source: Int J Mol Sci. 2025 Jul 25;26(15):7203. doi: 10.3390/ijms26157203 (PMC12346816; doi:10.3390/ijms26157203)
Supplement: Supplementary file 1 [file ijms-26-07203-s001.zip › ijms-3740973-supplementary.pdf]

Supplementary Materials

Assessment of Hydroxyl Radical Reactivity in Sulfur-Containing Amino Acid Models Under Acidic pH

Chryssostomos Chatgilialoglu, Piotr Filipiak, Tomasz Szreder, Ireneusz Janik, Gordon L. Hug, Magdalena Grzelak, Franciszek Kazmierczak, Jerzy Smorawinski, Krzysztof Bobrowski,* and Bronislaw Marciniak*

Correspondence: marcinia@amu.edu.pl (B.M.), kris@ichtj.pl (K.B.)

Contents pages

Figure S1 S2

Table S1 S2

Figure S2 S3

Figure S3 S4

Table S2 S4

Figure S4 S5

Figure S5 S5

Figure S6 S6

Figure S7 S6

Figure S8 S7

Figure S9 S7

Figure S10 S8

Figure S11 S8

Figure S12 S9

Figure S13 S9

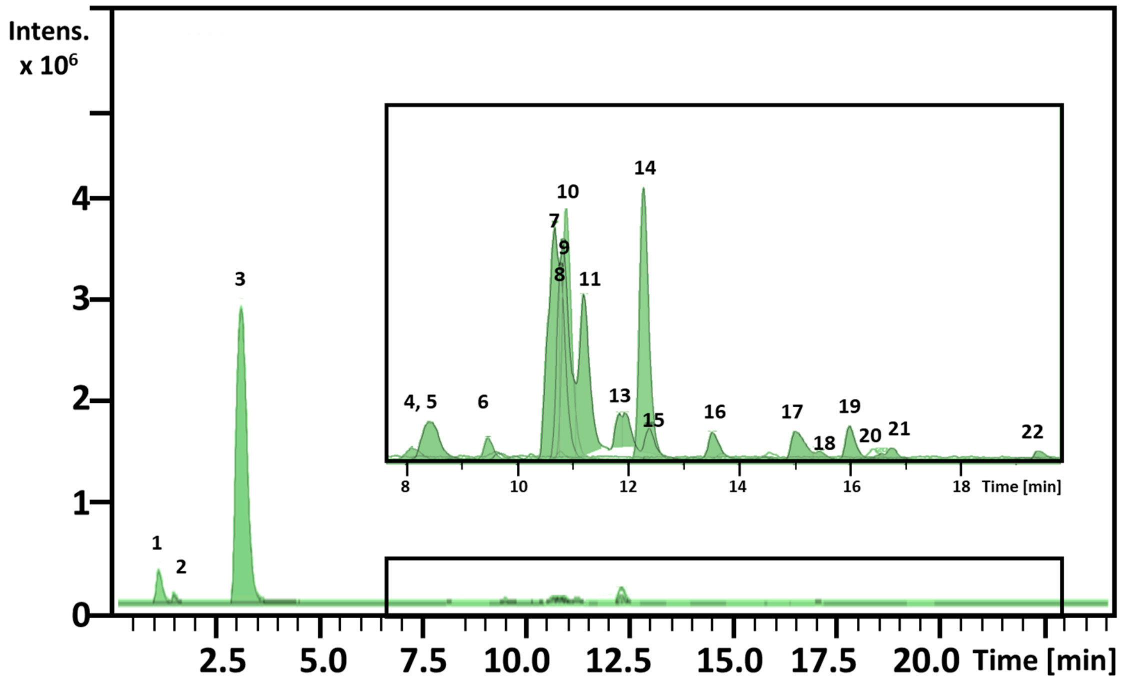

Figure S1. HPLC run of the crude reaction mixture of γ-irradiated N_2O-saturated aqueous solutions containing 1.0 mM of methionine derivative 1 (peak 3) at pH 4 at a dose of 800 Gy (dose rate of 46.7 Gy min−1); the consumption of 1 lead to the formation of 21 products. Inset: expansion of the chromatogram between 8 and 21 min.

Table S1. Conversion of Met derivative **1** (peak 3) after irradiation at 800 Gy at pH 4. The peaks 4-22 are shown in Figure S3

Peak	RT [min][b]	Peak Intensity	MH^+, *m/z*
1	1.1	319150	221.0954
2	1.5	83534	159.1128
3[a]	3.1	2885773	205.1005
4	8.1	3179	251.0882
5	8.4	9844	407.1781
6	9.5	5754	251.0882
7	10.7	60230	407.1781
8	10.8	49581	251.0882
9	10.9	55709	407.1781
10	10.9	65234	237.0726

11	11.2	42834	407.1781
12	11.9	12094	407.1781
13	11.9	12266	407.1781
14	12.3	70461	251.0882
15	12.4	8304	407.1781
16	13.5	7249	407.1781
17	15.0	7404	407.1781
18	15.4	2286	407.1781
19	16.0	8855	407.1781
20	16.6	1814	407.1781
21	16.8	3139	407.1781
22	19.4	2525	407.1781

[a] Starting material (Met derivative **1**); [b] retention time; [c] relative values.

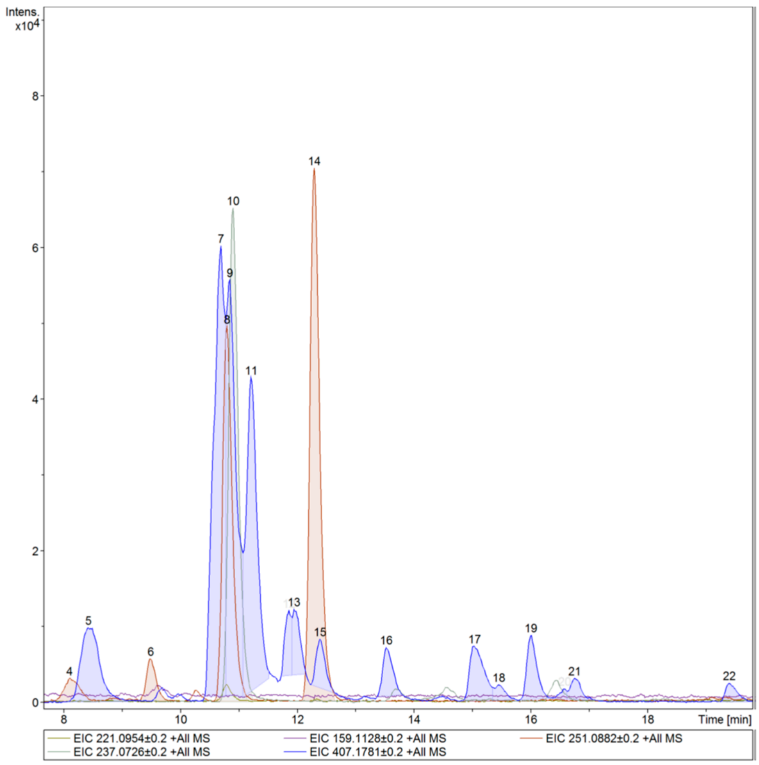

Figure S2. Fragments of LC-MS analysis for Met-containing derivative **1** after irradiation at 800 Gy at pH 4.

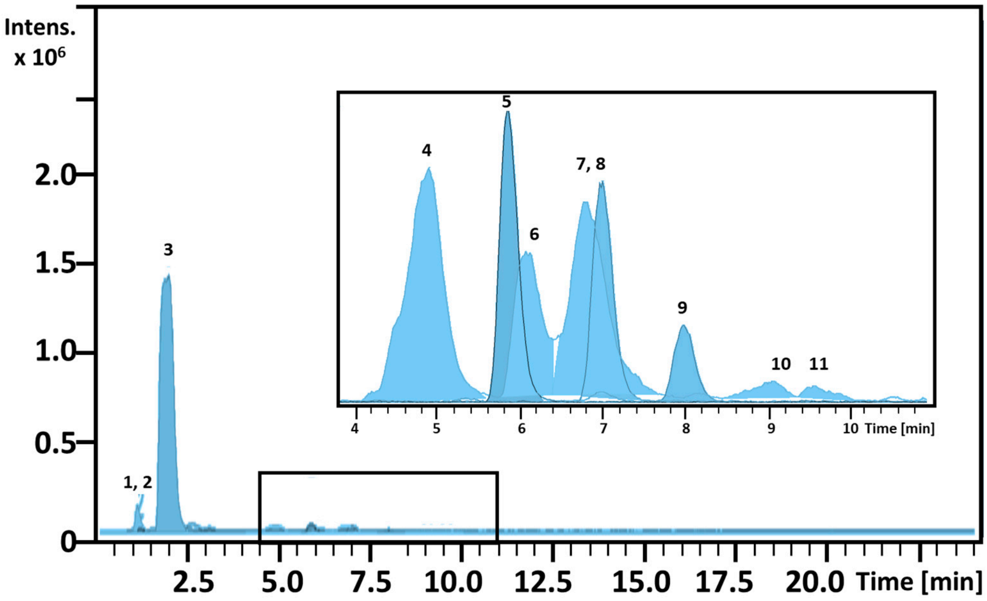

Figure S3. HPLC run of the crude reaction mixture of γ-irradiated N_2O-saturated aqueous solutions containing 1.0 mM of Cys-Me derivative 2 (peak 3) at pH 4 at a dose of 800 Gy (dose rate of 46.7 Gy min−1); the consumption of 2 lead to the formation of 10 products. Inset: expansion of the chromatogram between 4 and 11 min.

Table S2. Conversion of Cys-Me derivative **2** (peak 3) after irradiation at 800 Gy at pH 4. The peaks 4-11 are shown in Figure S6.

Peak	RT [min][b]	Peak Intensity	MH+, m/z
1	1.1	152029	207.0798
2	1.2	21510	145.0972
3[a]	1.9	1441767	191.0849
4	4.9	34978	379.1468
5	5.9	43830	223.0570
6	6.1	22595	379.1468
7	6.8	30228	379.1468
8	7.0	33026	237.0706
9	8.0	11659	237.0706
10	9.0	3355	379.1468
11	9.5	2701	379.1468

[a] Starting material (Cys-Me derivative **2**); [b] retention time; [c] relative values

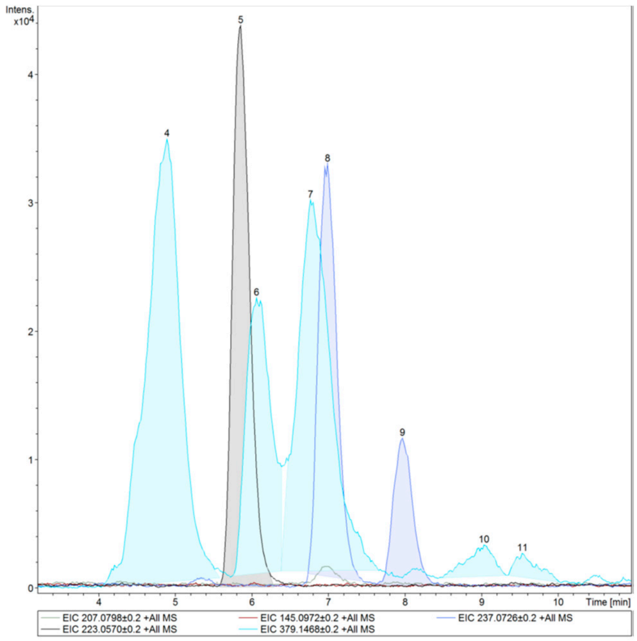

Figure S4. Fragment of LC-MS analysis for Cys-Me derivative **2** (peak 3) after irradiation at 800Gy in pH 4

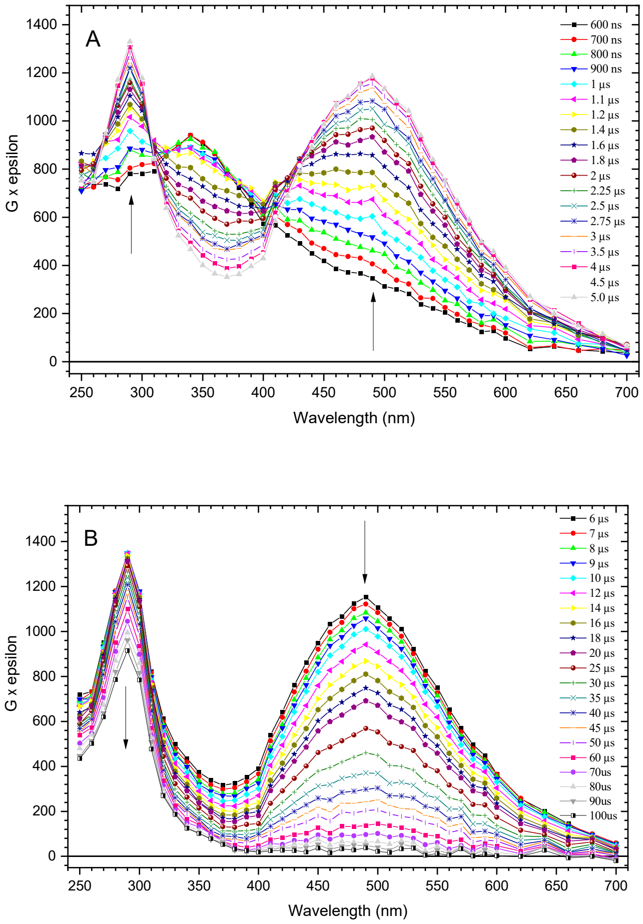

Figure S5 (A and B). Absorption spectra of N_2O-saturated aqueous solution of 0.2 mM of 1 at pH 4 recorded during pulse radiolysis after various time delays.

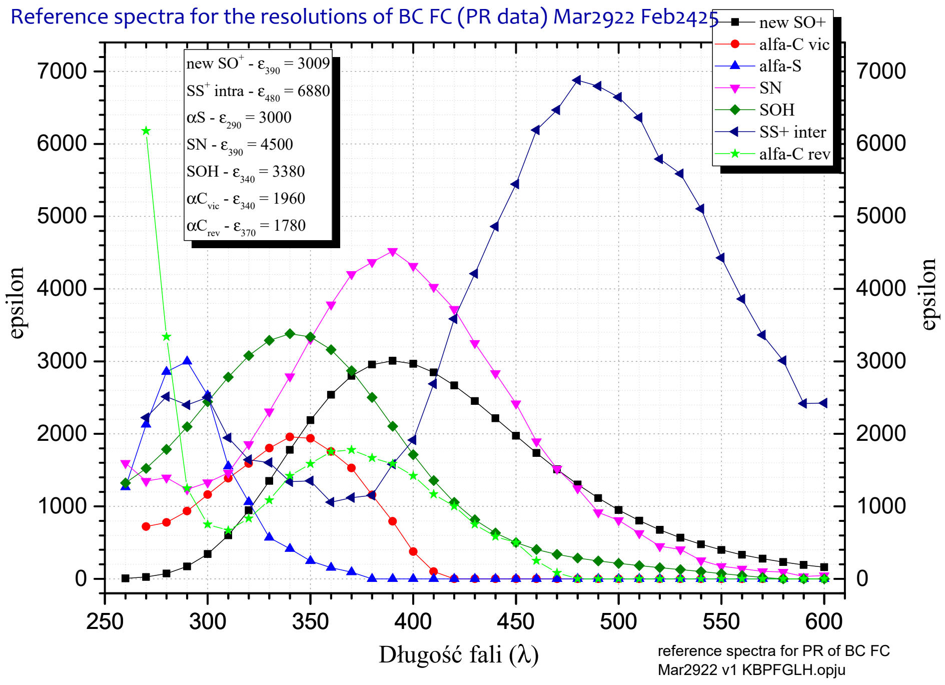

Figure S6. Reference spectra used in the resolution of the transient absorption spectra following •OH-induced oxidation of **1** and **2 (from ref. 23)**.

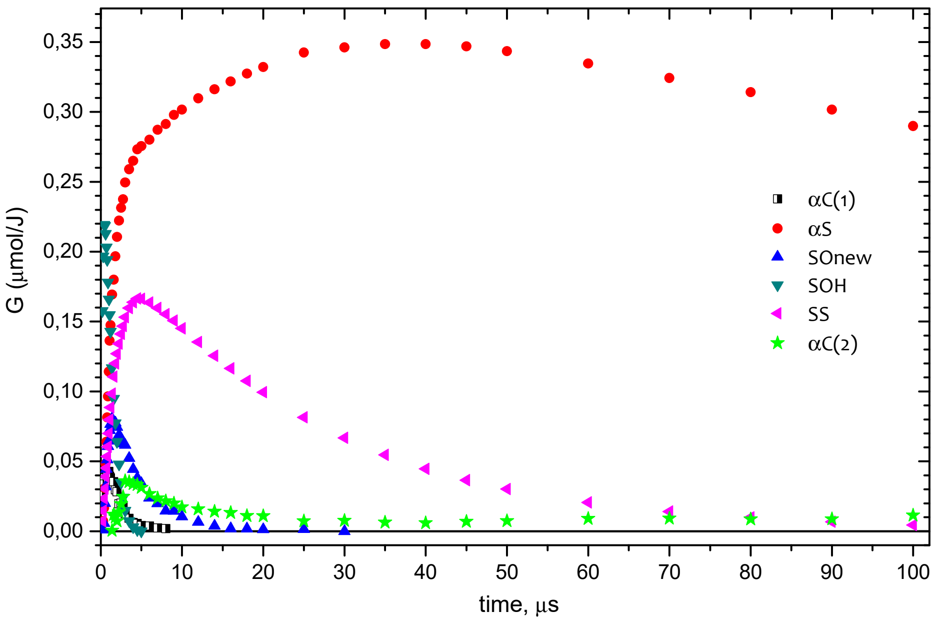

Figure S7. Concentration profiles of intermediates formed in the reaction of HO• with **1** obtained using spectral resolution and with the assumption of SO•+ contribution: aS• (●), SS•+ (◀), SO•+ (▲), HOS• (▼), αC(1)•(■), αC(2)• (★) in the time range up to 100 µs.

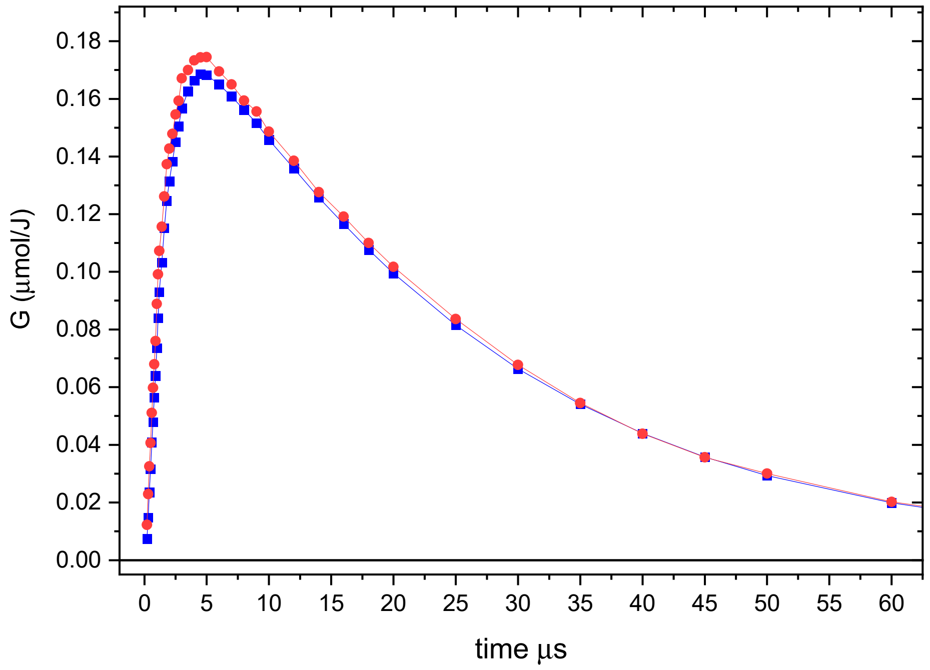

Figure S8. Concentration profiles of $SS^{•+}$ for **1** at pH 4 obtained from the spectral resolution (■) and from the absorbance at 490 nm (Figure 7A and B) taking ε = 6880 $M^{-1}cm^{-1}$ for $SS^{•+}$ (•).

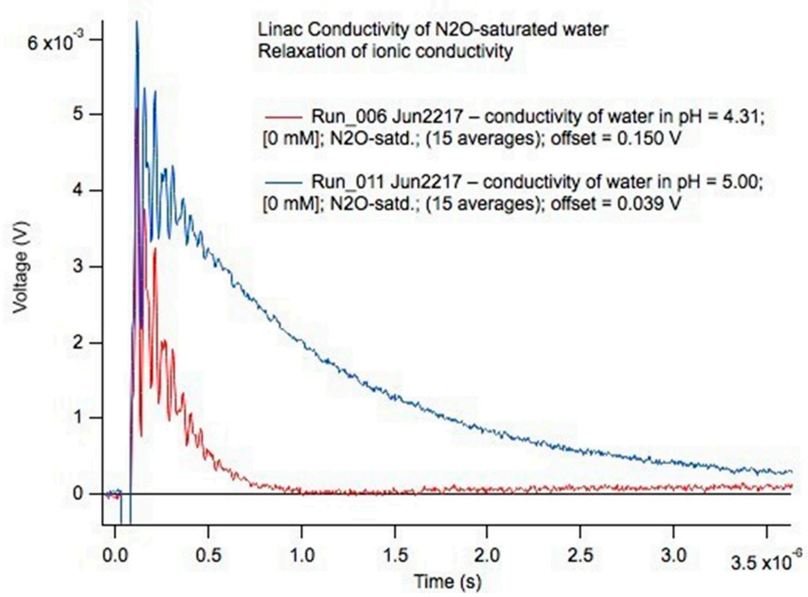

Figure S9. Relaxation of ionic conductivity after pulse radiolysis with conductivity detection of N_2O-saturated water at pH 4 (red) and pH 5 (blue).

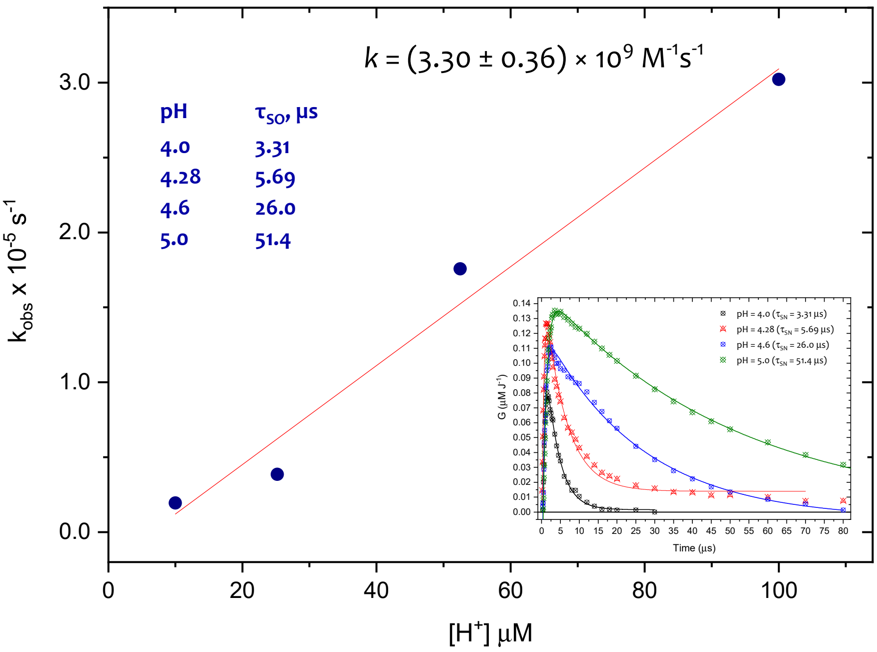

Figure S10. Dependence of k_{obs} for the decay of $SO^{\bullet+}$ on $[H^+]$ concentration. Inset: Concentration profiles for $SO^{\bullet+}$ represented as radiation chemical yields (G) vs time at various pH values: 4.0 (○), 4.3 (Δ), 4.6 (□), and 5.0 (⊗). Decays of $SO^{\bullet+}$ were fitted using the formula $G = G_0 \exp(-k_{obs}t) + G_{lim}$.

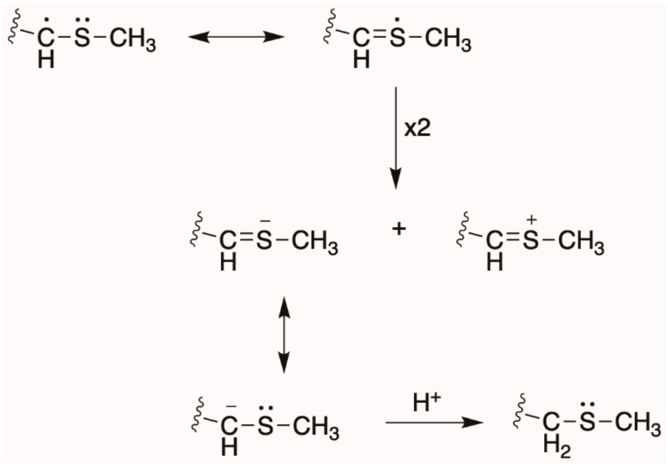

Figure S11. Reaction scheme presenting disproportionation of $\alpha S^{\bullet}$ derived from **1** or **2** in aqueous solution, on the example of $\alpha S^{\bullet}$ (2) derived from **1**, i.e., restoration of **1** and formation of the respective sulfur cation (S^+).

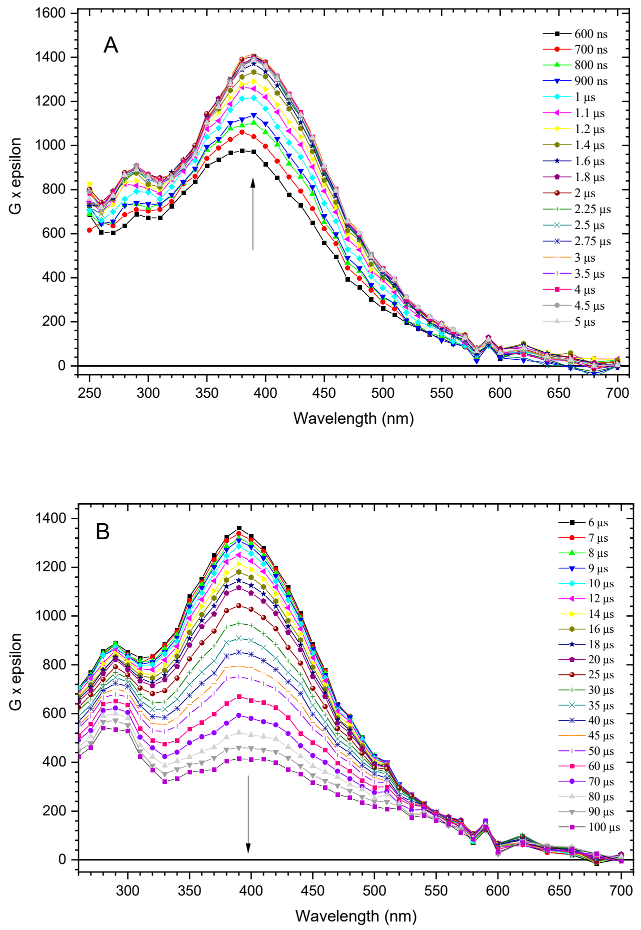

Figure S12 (A and B). Absorption spectra of N_2O-saturated aqueous solution of 0.2 mM of **2** at pH 4 recorded during pulse radiolysis after various time delays

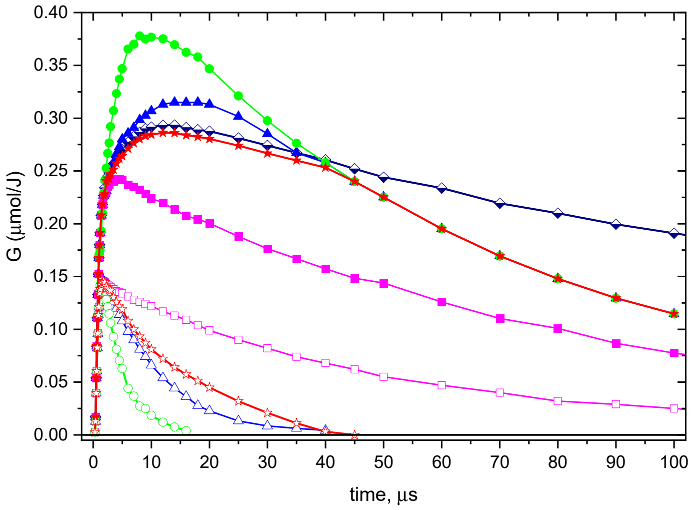

Figure S13. Concentration profile represented as *G*-values of ions formed in the reaction of $HO^{\bullet}$ with **2** obtained by division of $G \times \Delta\Lambda$ (*vide* Figure 12D) by the overall loss of equivalent conductivity (●); concentration profiles represented as *G*-values of $SO^{\bullet+}$ (●,▲,★,■) assuming $t_{1/2}$ = 3.4 µs (○), 9.6 µs (△), 13.4 µs (☆) and 48 µs (□) for $SN^{\bullet}$ decay, respectively.
